# New insights into the pathogenesis and transmission of *Brucella pinnipedialis*: systemic infection in two bottlenose dolphins (*Tursiops truncatus*)

**DOI:** 10.1128/spectrum.01997-23

**Published:** 2023-10-06

**Authors:** Ignacio Vargas-Castro, José Luis Crespo-Picazo, Manena Fayos, María de los Ángeles Jiménez-Martínez, Laura Torre-Fuentes, Julio Álvarez, André E. Moura, Marta Hernández, Aranzazu Buendía, Sandra Barroso-Arévalo, Teresa García-Seco, Marta Pérez-Sancho, María Jesús De Miguel, Sara Andrés-Barranco, Vicente Marco-Cabedo, Gaizka Peñin-Villahoz, Pilar María Muñoz, Lucas Domínguez, Daniel García-Párraga, José Manuel Sánchez-Vizcaíno

**Affiliations:** 1 VISAVET Health Surveillance Centre, Complutense University of Madrid, Madrid, Spain; 2 Animal Health Department, Veterinary School, Complutense University of Madrid, Madrid, Spain; ^3^ Fundación Oceanogràfic. Oceanogràfic. Ciudad de las Artes y las Ciencias, Valencia, Spain; 4 Centro de Recuperación de Fauna Silvestre de Cantabria, Santander, Spain; 5 Department of Animal Medicine and Surgery, Veterinary Faculty, Complutense University of Madrid, Madrid, Spain; 6 Museum and Institute of Zoology, Polish Academy of Sciences, Warsaw, Poland; 7 Laboratory of Molecular Biology and Microbiology, Instituto Tecnológico Agrario de Castilla y León, Valladolid, Spain; 8 Departamento de Ciencia Animal, Centro de Investigación y Tecnología Agroalimentaria de Aragón (CITA) - Instituto Agroalimentario de Aragón-IA2 (CITA-Universidad de Zaragoza), Zaragoza, España, Spain; CHU Grenoble, Grenoble, France

**Keywords:** milk, *Brucella pinnipedialis*, bottlenose dolphin, marine mammals, systemic infection, health surveillance, transmission, WGS

## Abstract

The emergence of *Brucella* infections in marine mammals is a growing concern. The present study reports two cases of systemic *Brucella pinnipedialis* infection detected in bottlenose dolphins (*Tursiops truncatus*) pair stranded together in the Cantabrian coast of Spain. Both animals showed systemic lesions associated with the *Brucella* infection, more severe in the younger dolphin, considered the likely offspring of the other individual. Real-time PCR, bacterial culture, and whole-genome sequencing were used to detect and characterize the *Brucella* strains involved in both dolphins. The phylogenetic analysis performed on the *Brucella* genomes retrieved revealed that the species involved was *B. pinnipedialis* (ST25). Both animals resulted seropositive in a commercial multispecies blocking ELISA but tested negative in the standard Rose Bengal test. To the best of our knowledge, this is the first report of a systemic infection resulting in various lesions associated with *Brucella pinnipedialis* (ST25) in two bottlenose dolphins. It is also the initial isolation of *Brucella* in the milk of a non-pregnant or non-aborting female cetacean likely stranded with its offspring. These findings provide new insights into the epidemiology and clinical impact of *B. pinnipedialis* infection in cetaceans and underscore the importance of continued diagnostic surveillance to gain better understanding of brucellosis effects and transmission in marine mammal populations.

**IMPORTANCE:**

*Brucella* spp. are zoonotic pathogens that can affect both terrestrial and marine mammals. *Brucella ceti* has been identified in various cetacean species, but only one sequence type (ST27) has been reported in humans. However, it is important to conduct surveillance studies to better understand the impact of marine *Brucella* species on marine mammals, a typically understudied host group. Here, we describe a systemic infection by two related strains of *Brucella pinnipedialis* (ST25) in a couple of live-stranded bottlenose dolphins, with more severe lesions in the younger animal. Furthermore, *B. pinnipedialis* was first detected in milk from a female cetacean that stranded with its offspring. Our study reveals novel insights into the epidemiology and pathological consequences of *B. pinnipedialis* infections in cetaceans, emphasizing the crucial importance of ongoing surveillance and accurate diagnosis to understand the impact of this pathogen on marine mammal populations.

## INTRODUCTION

Bacteria of the genus *Brucella* spp. are Gram-negative, intracellular bacteria, mostly zoonotic, and can infect a wide range of hosts, including terrestrial and marine mammals (1). *Brucella* infections in marine mammals were first described in 1994, and since then, brucellosis has been reported in different species of cetaceans and pinnipeds (2, 3). In 2007, these *Brucella* species were splitted into two species: *Brucella ceti* and *Brucella pinnipedialis,* associated to cetacean and pinniped preferential hosts, respectively (4).

Brucellosis in cetaceans has been associated mainly to meningitis or meningoencephalitis (2, 5 – 16); placentitis, placental abscesses, abortions, and stillbirths (2, 8, 11 – 13, 17 – 19); epididymitis and orchitis (11, 20, 21); osteoarthritis, discospondylitis, and vertebral osteomyelitis (8, 11, 17, 22, 23); pneumonia and lung abscesses (14, 15, 17, 24); hepato-, spleno-, and lymphadenomegaly with necrotic foci and inflammation (14, 17), and blubber and sub-blubber abscesses (11, 25). In spite of this, it is very common that marine mammals diagnosed with *B. ceti* or *B. pinnipedialis* infection do not present pathological changes associated with brucellosis (26 – 28), which highlights the difficulties in the assessment of the clinical significance of *B. ceti* and *B. pinnipedialis* isolations from marine mammals. Laboratory diagnosis and bacteriological culture, in particular, are essential to confirm any *Brucella* infection (29). No serological test has been validated for the specific diagnosis of brucellosis in marine mammals, so those used for terrestrial animals are often applied to marine mammals (25, 30 – 33).

Direct detection of brucellosis has been reported in at least 16 species of cetaceans (34 – 38) throughout the world, while more than 50 species of marine mammals have shown serological suspicions of brucellosis (38). *Brucella* isolates from marine mammals have been clustered into five distinct sequence types (ST23, ST24, ST25, ST26, and ST27). Among them, the closely related ST24 and ST25 belong to *B. pinnipedialis* species and are primarily associated with seal isolates. The remaining three STs (ST23, ST26, and ST27) are linked to *B. ceti* and porpoise isolates (ST23), dolphin isolates (ST26), and both bottlenose dolphin and human isolates (ST27) (26, 39 – 42).

Brucellosis surveillance in marine mammals and further studies are needed to gain understanding in the impact of marine *Brucella* not only on public health but also on the monitoring programs of marine mammal populations.

To the best of our knowledge, the present paper describes for the first time two cases of systemic infection with lesions associated with *B. pinnipedialis* in two bottlenose dolphins and the first isolation of this bacteria in cetacean milk. Detection was done through the infectious disease surveillance program in the stranding network of the Cantabrian coast (Spain).

## RESULTS

### Macroscopic exam

The total length of dolphin Tt1 was 309 cm, whereas that of dolphin Tt2 was 197 cm. Main macroscopic findings included: Tt1 in good body condition, free blood in the thorax cavity, bloody lung parenchyma on section, heavy presence of gastric nematodes (*Anisakis* sp.), and seven recreational fishing hooks in the first gastric chamber. It presented milk on mammary glands on section. Tt2 had a good body condition, white foam and bloody lung parenchyma in section, milk content in the first gastric chamber, and severe brain congestion.

### Age estimation

The estimated age of Tt2 was 2.324–3.595 years with 95% confidence, so it was considered juvenile.

Since in the model used for this study, the predicted asymptotic length for females was 246 cm, and Tt1 measured 309 cm, her age could not be determined, but it was assumed that she was an adult due to her length and to the fact that she was lactating.

### Relatedness analysis

Allele sizes for the microsatellites are indicated in Table 1. Both individuals share one allele at every locus, with the second allele not being shared for most locus.

**TABLE 1:** Allele sizes for the microsatellites

	Tt1		Tt2	
	Allele 1	Allele 2	Allele 1	Allele 2
Dde84	157	145	145	145
Dde65	197	189	189	189
AAT44	86	80	86	86
Dde70	153	135	149	135
Ttr58	193	191	193	183
Dde69	207	207	207	207
Dde59	252	240	240	240
Ttr63	135	105	135	105
Ttr34	190	180	182	180
Dde66	357	357	357	357
TtrC12	125	113	125	111
KWM1b	188	184	188	184
KWM12a	178	168	178	166

The Queller and Goodnight index and the Lynch and Ritland index were 0.575 and 0.426, respectively.

### Histopathology

**Fig 1:**
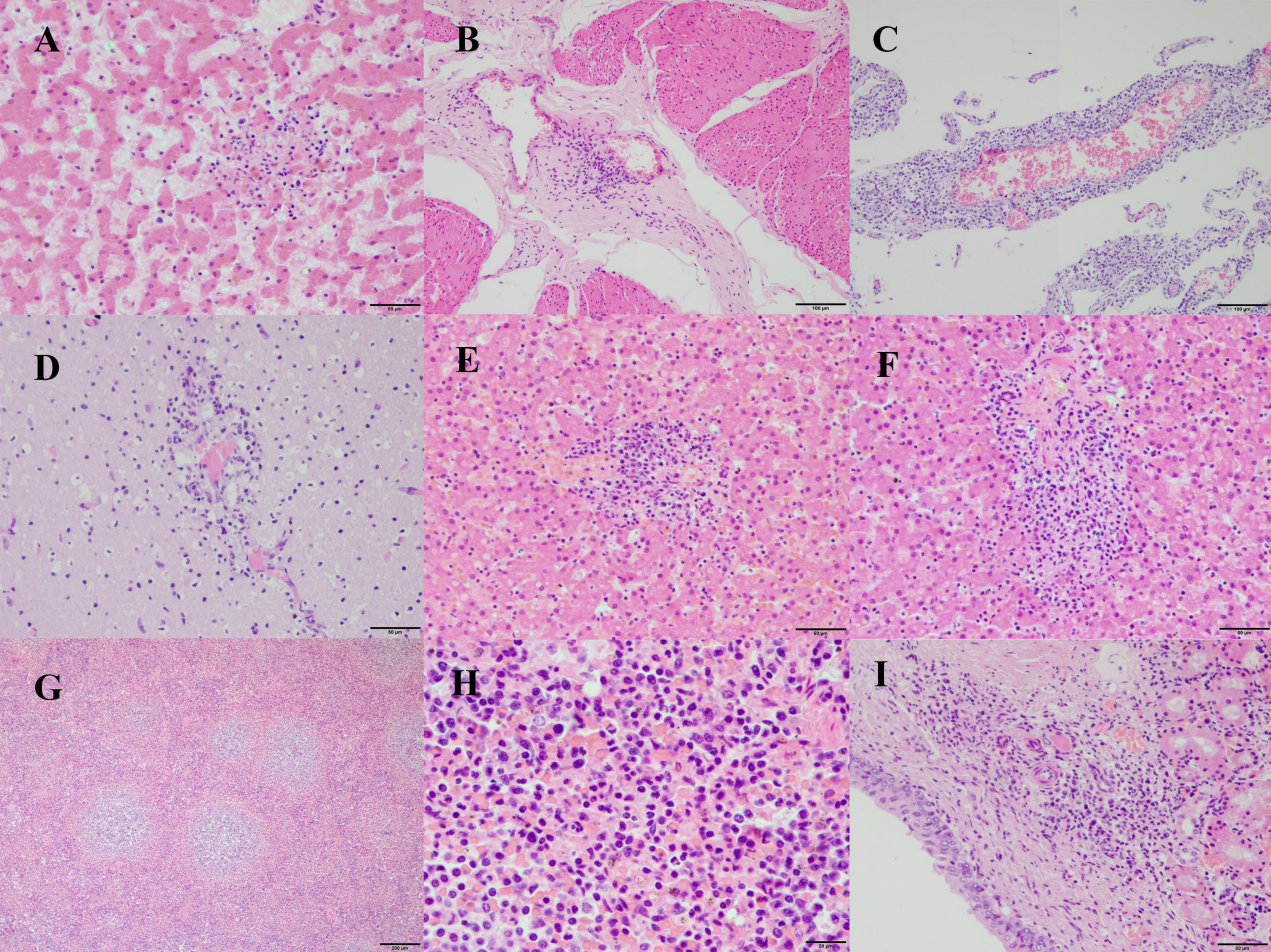
Main histopathological findings. Images A and B correspond to Tt1, while images C–I correspond to Tt2. (A) Perivascular and sinusoidal infiltrate of lymphocytes, histiocytes, plasma cells, and some neutrophils in the liver parenchyma. (B) Perivascular infiltrates of lymphocytes, histiocytes, and some neutrophils in the connective tissue between the muscular layers of the urinary bladder. (C) Markedly expanded meninges by extensive perivascular infiltrates of macrophages, lymphocytes, fewer neutrophils, and plasma cells. (D) Perivascular infiltrates of macrophages, lymphocytes, fewer neutrophils, and plasma cells in the neuropil, and numerous hypertrophied astrocytes. (E and F) Presence of nodular aggregates of lymphocytes, macrophages, fewer neutrophils, and plasma cells in the portal tracts and surrounding the centrilobular veins. The sinusoids are diffusely dotted with the same cells and moderately congested. Kupffer cells are hypertrophied. Hepatocytes contain small ill-defined colorless and eosinophilic vacuoles. (G) Diffusely, the follicles of the spleen are markedly enlarged and contain large germinal centers. (H) In the red pulp of the spleen, there are abundant extramedullary hematopoiesis, neutrophils, and some macrophages with hemosiderin inside. (I) Presence of foci of lymphocytes, macrophages, and neutrophils in the renal pelvis.

The main histopathological lesions observed in Tt1 were mild multifocal lymphoplasmacytic and suppurative hepatitis (Fig. 1A) with diffuse congestion, and fibrosing and lymphoplasmacytic multifocal mild-to-moderate cholangiohepatitis with biliary hyperplasia; mild multifocal lymphoplasmacytic and suppurative cystitis (Fig. 1B); minimal multifocal lymphoplasmacytic and histiocytic meningitis; mild confluent multifocal pyogranulomatous bronchopneumonia with intralesional metastrongyles; mild diffuse sinus histiocytic lymphadenitis in the lung pulmonary node; moderate confluent multifocal lymphonodular fibrosis in the mesenteric lymph node; and mild diffuse histiocytic and suppurative sinus lymphadenitis in the pre-scapular lymph node.

The main histopathological lesions observed in Tt2 were severe confluent multifocal pyogranulomatous meningoencephalitis (Fig. 1C and D); moderate multifocal pyogranulomatous pneumonia with severe edema and congestion; moderate-to-severe multifocal pyogranulomatous hepatitis (Fig. 1E and F); mild-to-moderate diffuse histiocytic and suppurative splenitis and extramedullary hematopoiesis (Fig. 1G and H); mild multifocal pyogranulomatous cystitis; mild-to-moderate multifocal pyogranulomatous pyelonephritis (Fig. 1I); moderate diffuse suppurative tonsillitis; mild-to-moderate diffuse histiocytic and suppurative lymphadenitis.

The histological descriptions of the *Brucella*-positive tissues in the molecular diagnosis are specified in more detail in Supplementary Material S2.

### Molecular diagnosis

Positive PCR results were obtained in milk, spleen, and urinary bladder samples from Tt1, and in central nervous system (CNS; brain, cerebellum, and spinal cord), cerebrospinal fluid (CSF), lymph nodes (mesenteric and pulmonary), lung, testis, and urinary bladder samples from Tt2 (Supplementary Material S2). BLAST analysis of the amplified DNA fragments confirmed the presence of DNA from *Brucella* spp. Due to the short length of the amplified fragment, after excluding the primers, the sequences obtained were not deposited in GenBank.

All samples analyzed were negative in the molecular diagnosis of CeMV infection.

### Bacterial culture and classification

The bacterial culture was positive for *Brucella* spp. in the milk of Tt1 and multiple organs including the cerebrum, lung, pulmonary lymph node, urinary bladder, and kidney of Tt2 (Supplementary Material S2). Bacterial typing using the method described below showed homology with *B. pinnipedialis* (B2/94).

### Phylogenetic analysis

Genomes from both samples were classified as multilocus sequence typing (MLST) sequence type 25 (ST25).

**Fig 2:**
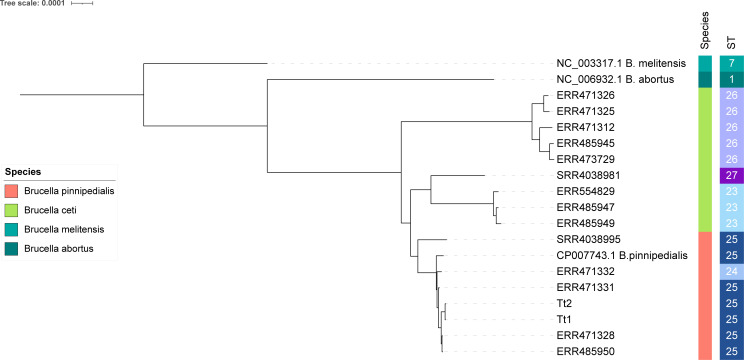
Maximum-likelihood phylogenetic tree. Strains sequenced in the present study are named Tt1 and Tt2, based on the host ID. External sequences of *Brucella* are named according to European Nucleotide Archive or Sequence Read Archive identifier. *Brucella* species and STs are indicated in the two bars on the right.

The constructed phylogeny showed two major clades separating *B. ceti* belonging to ST26 from a second clade that included *B. ceti* (ST27 and ST23) and *B. pinnipedialis* (ST25 and ST24) forming two separate subclades as previously described (14). Strains Tt1 and Tt2 clustered with *B. pinnipedialis* isolates belonging to ST25 (Fig. 2) and differed by 90–97 single nucleotide polymorphisms (SNPs) from the genetically closest strains, two *B. pinnipedialis* strains retrieved from a common seal and an otter in Scotland in 1994 (ERR471328, ERR485950). The comparison of Tt1 and Tt2 genomes revealed the presence of 18 high-quality SNPs between them.

### Serology

Both individuals were seropositive using the blocking ELISA, presenting a high percent inhibition (PI) (Tt1: 94.42%, Tt2: 94.62%). However, both animals were negative in the Rose Bengal test (RBT).

## DISCUSSION

Brucellosis is a bacterial zoonotic disease that affects a wide range of mammalian species, including marine mammals. Specifically, *B. pinnipedialis* has been considered a potentially zoonotic pathogen (1, 38). In this study, we report a case of *Brucella* infection in two bottlenose dolphins stranded on the Cantabrian coast of Spain. The diagnosis was confirmed by PCR and bacterial culture, identifying the species involved as *B. pinnipedialis* ST25 based on molecular typing and whole-genome sequencing (WGS) analysis. Furthermore, both animals had detectable *Brucella* spp.-specific antibodies according to a blocking ELISA despite a negative RBT result. The histopathological study revealed systemic lesions associated with *Brucella*, with particularly severe inflammation observed in multiple Tt2 organs.

The age estimation of the two bottlenose dolphins concluded that they were a juvenile (Tt2) and an adult (Tt1), which is consistent with the age estimation from total length that Geraci and Lounsbury established for bottlenose dolphins (43). Both animals stranded together, and they shared one allele at every *locus*, with the second allele not being shared for most *loci*. Moreover, their relatedness indexes were around 0.5 (LR index was multiplied by 2), which is consistent with a parent-offspring pairing (44, 45). Given the collective evidence presented above, both individuals were considered as a likely mother and offspring pair.

In both individuals, a *Brucella* spp. infection was revealed through molecular detection and bacterial culture (Supplementary Material S2). Although bacterial isolation is considered the “gold standard” for the diagnosis of *Brucella* infections, real-time PCR from purified DNA directly extracted from tissues or fluids has also demonstrated its value as an effective diagnostic tool (46, 47), and it has previously been used to diagnose brucellosis in cetaceans (9, 48). The phylogenetic analysis conducted on the sequenced genomes classified both isolates as ST25 *B. pinnipedialis* (Fig. 2), which is consistent with the bacterial classification analysis. However, SNP typing revealed 18 SNPs between both strains, which suggest that the infection observed in the mother and the offspring may have originated from different sources. In some bacteria, such as *Pseudomonas aeruginosa*, isolates with up to 26 SNPs differences are considered to have epidemiological linkage (49). On the other hand, in *B. melitensis*, a seven-SNP threshold is applied to detect the clusters of closely related cases (50). To the best of the authors’ knowledge, an SNP threshold for distinguishing marine *Brucella* strains has not yet been established. Applying the same threshold used for *B. melitensis* to this case would suggest that the two sequences correspond to different strains of *B. pinnipedialis*. However, it cannot be ruled out that they were infected by the same strain since both animals presented a similar course of the disease and were likely a mother/offspring pair, with the female lactating, the pathogen isolated from her milk, and the presence of milk detected in the first gastric chamber of the younger animal. Further information on the genetic variability between field strains of marine *Brucella* (*B. ceti* or *B. pinnipedialis*) may help to establish SNP thresholds to differentiate between epidemiologically related strains in the future.

The pathological study revealed that Tt1 had a mild-to-moderate systemic infection, mainly affecting the liver and urinary bladder, with vascular and perivascular tropism. This predominant vascular distribution of inflammation and the mixed infiltrate were consistent with a systemic *Brucella* spp. infection (5). In this case, the CNS was not significantly affected. In contrast, Tt2 had a systemic inflammatory process that severely affected the meninges and brain, liver, spleen, kidney, and lung. The fact that the lesions were more severe in Tt2 is especially striking, since in terrestrial mammals, young animals are usually resistant to infection compared to sexually mature animals or pregnant females (51, 52). In cetaceans, however, several cases of *Brucella*-associated lesions have been reported in young individuals, mainly juveniles or subadults (9, 14). Other factors, such as the dose and virulence of the infecting strain, may also play an important role, as previously suggested (52). Future studies should address the impact of the age and other risk factors on the pathological manifestations of brucellosis in cetaceans.

Meninges and brain involvement are characteristics of cetacean brucellosis (26), and the central nervous system is the most affected in these animals (2, 5 – 15). Differently, this study outlines two systemic infections, with severe inflammation in various organs in Tt2. The presence of inflammation, predominantly in the liver, spleen, and lung, is consistent with the systemic phase of infection observed when this agent infected other species (52). However, the pathogenesis and tissue and organ dissemination of this agent in cetacean hosts are not fully understood (14). In other terrestrial animal species, *Brucella* infections are initially systemic. In the acute phase, the bacteria extend quickly to the regional lymph nodes, causing acute lymphadenitis there. The infection may be overcome in the regional lymph nodes, or it may spread hematogenously, and bacteremia may persist, with consequent systemic infection (52, 53). An alternative hypothesis that cannot be ruled out is that the infection was latent and reactivated, a phenomenon that has been suggested to occur in marine mammals (54).

Another interesting finding was the detection of *Brucella* in the female’s milk without evidence of infection in the reproductive tract. Unfortunately, samples from the mammary glands were not available in this study, but bacterial presence in this location cannot be ruled out. The inclusion of mammary tissue in future studies should be encouraged. *B. abortus* is capable of infecting the pregnant uterus but does not persist well in the non-pregnant uterus. However, in bacteremic episodes, the bacteria can be localized and persist in multiple tissues, including the mammary glands (52). If *B. pinnipedialis* showed similar pathogenesis, it would explain why Tt1 had a negative result in uterus samples but positive in the milk. Nevertheless, further studies are necessary to confirm this hypothesis. On the other hand, in other hosts such as sheep, the elimination of *B. melitensis* in milk has been described at least up to 125 weeks post-infection, even in the absence of clinical signs (55). In cetaceans, the presence of *B. ceti* has been described in fetal tissues, secretions, and milk of a pregnant striped dolphin female (12). Also, *Brucella* spp. infection in milk has been described in other cetacean species, both in generalized infections (11, 19) and cases in which the mammary gland or milk was the sole site of infection (37, 40). Being a reproductive disease, brucellosis can have a great impact on the population dynamics of cetaceans (26), although its real importance at this level remains unknown (13).

Unfortunately, it is not possible to determine the route of infection. In spite of the genetic similarity found between strains, the differences between their sequences do not allow to conclude if they were infected by the same strain (which would be likely transmitted from mother to offspring) or if they were infected by two genetically related yet different strains from two different sources. The infection could have occurred horizontally, through maternal feeding, as described for other terrestrial species (53) and as it has been suggested also in cetaceans (26). In fact, Tt1 and Tt2 stranded in March, coinciding with the start of the calving season of this species in the northern hemisphere, which has been associated with a peak of brucellosis prevalence in bottlenose dolphins (56). In this context, it has been previously suggested that the transmission of *Brucella* in marine mammals increases during the spring, when calves are nursing (26, 56). Although Tt2 is considered a juvenile, milk was found in its first gastric chamber. It has been reported that the lactating period of bottlenose dolphins under human care can last up to 37 months (57) and 3.2 ± 0.6 years on average in the wild (58). The age of Tt2 is estimated to be between 2.324 and 3.595 years old, suggesting that he may have still been nursing. However, there is no certainty that Tt2 contracted the infection through maternal feeding. Nevertheless, to the best of the authors’ knowledge, this is the first reported case in which *Brucella* presence in milk occurs in a non-pregnant or non-aborting female stranded along with its offspring. This suggests that the mammary gland could be a persistent infection site also in dolphins, reinforcing the idea that *Brucella* spp. also have a tropism for the udder in cetaceans, as previously proposed (26). Consequently, milk could represent a transmission source for newborns and calves.

In *B. ceti,* a link between phylogeny and topographical distribution has been suggested (14). The first description of brucellosis in a cetacean species (striped dolphin) in Spain also occurred in the Cantabrian region, although the causative agent implicated was identified as *B. ceti* (6). In this case, we describe an infection by *B. pinnipedialis*, ST25, in two bottlenose dolphins stranded in the same region. Despite *B. pinnipedialis* being usually associated with infections in different seal species (4), there are some reports of infections in cetaceans, involving both Odontocetes and Mysticetes, mostly associated with ST24 (16, 40, 42, 59) but also with ST25 (42, 60). Furthermore, *B. pinnipedialis* isolates from pinnipeds are usually associated with infections in clinically healthy animals without associated pathologies (38, 39). In cetaceans, there is only a single documented case of pathology associated with *B. pinnipedialis*, which consisted of neurobrucellosis in a common minke whale (16). In contrast, both Tt1 and Tt2 reported here presented a systemic infection. Tt1 exhibited mild-to-moderate infection, primarily affecting the liver and urinary bladder, while the CNS was not significantly affected. Conversely, Tt2 presented a severe systemic infection that significantly compromised multiple organs, including the meninges, brain, liver, spleen, kidney, and lung.

The determination of antibodies against *Brucella* spp. was negative for RBT and positive for blocking ELISA. Similar results (RBT−, blocking ELISA+) have been obtained in 7 out of 10 individuals (70%) of different species of cetaceans, including a bottlenose dolphin (31) and in different Antarctic pinniped species (61). The serum samples used in our study were highly hemolyzed and were frozen before performing the tests, which is not recommended when performing the RBT, according to manufacturer’s instruction. Thus, the use of samples that are not fresh and/or that are hemolyzed may explain the negative results obtained with the test. However, hemolyzed samples often give false-positive results (26, 62), whereas false-negative results usually correspond to a low overall avidity or low titers of binding antibodies (62). On the other hand, in the absence of a gold-standard test, the use of competitive or blocking ELISA has been considered an appropriate option to detect antibodies against *Brucella* spp. in marine mammals, not only because it is a multispecies test but also because it can be used in low-quality samples, which is the most common scenario in these species (30 – 33, 61, 63 – 67). Therefore, in our study, we have considered positive blocking ELISA results as an indicative of the presence of antibodies against *Brucella*, as previously reported (61). These results emphasize the importance of validating different diagnostic techniques for accurate direct or indirect detection of *Brucella* infections in cetaceans.

In conclusion, our findings add evidence to a growing body of literature on marine brucellosis and open new avenues of investigation that should be explored in the future. To the authors’ knowledge, this is the first time that a systemic infection with different lesions associated with *Brucella pinnipedialis* (ST25) has been described in two bottlenose dolphins, providing new information on the pathogenesis of this bacteria in cetaceans. The positive isolation of *Brucella* in a milk sample from a non-pregnant or non-aborting female cetacean stranded alongside its offspring is described for the first time, suggesting that the mammary gland could be a persistent infection site in dolphins and milk could serve as a transmission source. Furthermore, this study raises new questions that should be explored in new research. This includes establishing an SNP threshold to determine whether marine *Brucella* isolates may be epidemiologically linked in WGS-based studies, the pathological importance of *B. pinnipedialis* in cetacean populations, and the likelihood of *B. pinnipedialis* transmission between cetaceans and, if epidemiologically relevant, through which routes this transmission occurs.

## MATERIALS AND METHODS

### Naturally infected dolphins

The bottlenose dolphins (*Tursiops truncatus*), Tt1 and Tt2, included in the present study stranded alive in Oyambre Beach, Cantabria, Spain (43°23′34″N, 4°20′03″ W) on 5 March 2020. The individual Tt2 was dead at the time of first response, while the individual Tt1 was alive and was subsequently humanely euthanized. Dolphin Tt1 was a female and dolphin Tt2 was a male. Body condition was good in both animals, and the dolphin carcasses were fresh at the time of examination.

Detailed necropsies of both animals were carried out as described previously (43, 68). During the necropsies, the following tissues were sampled for molecular diagnosis and bacterial culture: skin, muscle, blubber, cerebrum, cerebellum, spinal cord, lymph nodes (mesenteric, pre-scapular, and pulmonary), lung, rete mirabile, gonad, liver, kidney, urinary bladder, heart, and spleen. Samples from the uterus and milk from Tt1 and samples from thyroid, cerebrospinal fluid, and epididymis from Tt2 were also obtained.

For the histopathological study, samples of cerebrum, lung, pulmonary lymph node, liver, kidney, urinary bladder, skeletal muscle, skin, and blubber were taken. Additionally, samples were taken from mesenteric and pre-scapular lymph nodes and ovary from Tt1; and spleen, pharyngeal tonsils, pre-scapular lymph node, myocardium, and testicle from Tt2.

The set of samples for molecular diagnostics was stored at −80°C, while the set of samples for conventional histopathology was preserved in 10% neutral buffered formalin.

Serum samples with high degree of hemolysis were also obtained and kept frozen for the serological study.

### Age estimation

The age of both individuals was estimated with a 95% confidence interval following Gompertz model (69):

where *L*_*t*_ = total length at age *t*, *L*_0_ = total length at age 0, *G* = initial growth rate, and *g* = rate of exponential decay of growth rate, using the parameter values developed previously for bottlenose dolphins (70).

### Relatedness analysis

A total of 13 microsatellite *loci* were amplified through two multiplex PCR reactions using Qiagen Type-it Microsatellite PCR Kit (Qiagen, Hilden, Germany). Each multiplex reaction included primers that amplify microsatellite *loci* across cetacean species, with the following conditions: 95°C for 15 min, 40 cycles at 52°C (primer set A)/57°C (primer set B) for 90 s, 72°C for 1 min, and a final extension at 60°C for 30 min. Primer set A amplified *loci* Dde66, TtrC12, KWM1b, and KWM12a. Primer set B amplified *loci* Dde59, Dde65, Dde69, Dde70, Dde84, AAT44, Ttr19, Ttr58, and Ttr63 (71). Microsatellite allele sizes were determined through visual inspection of capillary electrophoresis traces (ABI 3500 genetic analyzer) in Geneious R7. The analyses were replicated twice for each individual, and final genotypes were determined by consensus between the two replicates.

Relatedness score between the two individuals was determined using the Queller and Goodnight (44), and Lynch and Ritland [(45); multiplied by 2 to give a range of −1 to 1] indexes, using GenalEx 6.5 (72).

### Histology

Samples fixed in 10% neutral buffered formalin were embedded in paraffin, sectioned at 4 ± 2 µm, stained with hematoxylin and eosin, following routine laboratory procedures, and examined through a light microscope by a certified veterinary pathologist.

### Molecular diagnosis

The Tt1 and Tt2 cases included in the present study were diagnosed during the routine health surveillance carried out on the stranded dolphins in Cantabria. This health surveillance has been proposed as a good tool to increase the probability of early detection of disease outbreaks caused by agents such as *Brucella* spp. and cetacean morbillivirus (CeMV) (15). The molecular detection of *Brucella* was carried out because it is a cause of mortality in these animals and because of its zoonotic potential, and CeMV presence was analyzed due to the high mortality that this virus can produce in cetacean populations.

All samples were diluted 1:10 with phosphate-buffered saline and homogenized using stainless steel 4.8 mm beads (Next Advance, New York, USA). RNA and DNA from the homogenates were extracted using the High Pure Viral Nucleic Acid Kit (Roche Diagnostics, Mannheim, Germany), based on the manufacturer’s instructions.

For *Brucella* spp. molecular diagnosis, a previously described real-time PCR targeting the insertion sequence IS*711* was performed (73) in the following tissue samples: cerebrum, cerebellum, spinal cord, CSF lymph nodes (pulmonary and mesenteric), lung, kidney, urinary bladder, ovary/testicle, uterus, and milk. *Brucella melitensis* vaccine strain B115 DNA was used as positive control, while ultrapure water was used as negative control.

Nucleic acid extracts from cerebrum, cerebellum, pulmonary and pre-scapular lymph nodes, lung, kidney, and cerebrospinal fluid were assayed for CeMV using a reverse transcription PCR method based on the Universal Probe Library platform that amplifies the fusion protein gene (74). CeMV-positive striped dolphin (*Stenella coeruleoalba*) pharyngeal tonsils RNA was used as positive control, while ultrapure water was used as negative control.

Positive PCR products were purified using the QIAquick PCR Purification Kit (Qiagen, Hilden, Germany), amplicons were sequenced completely by Sanger sequencing, and sequences were compared with known GenBank sequences by using BLAST.

### Bacterial culture and classification

Different tissue samples from Tt1 (cerebrum, lung, pulmonary lymph node, milk, CSF, uterus, kidney, and urinary bladder) and Tt2 (cerebrum, lung, pulmonary lymph node, testicle, kidney, and urinary bladder) were processed following the protocol described elsewhere (75). Briefly, samples were degreased, superficially sterilized by gentle burning, and homogenized in a minimal amount of buffer using a Stomacher. Cultures of at least 0.5 mL of each homogenate or fluid (such as cerebrospinal fluid, milk, or urine) were plated in duplicate on CITA and Farrell selective media and incubated for 5–7 days at 37°C in a 10% CO_2_ atmosphere. Suspicious colonies were identified as *Brucella* using standard procedures (76) and the Bruce-ladder multiplex PCR (77), which enables the identification of the main *Brucella* species, including those that affect marine mammals. Bacterial DNA was extracted using the Speedtools Tissue DNA Extraction Kit (Biotools, Madrid, Spain).

To differentiate between *B. ceti* and *B. pinnipedialis*, a multiplex PCR adapted from López-Goñi et al. (78) was used, employing the following two pairs of primers: T TCA ACT GCG TGA ACA ATG CT (f)/GCG GGC TCT ATC TCA AGG TC (r), and CGT CAA CTC GCT GGC CAA GAG (f)/GCA GGA GAA CCG CAA CCT AA (r). All isolates were also typed by PCR-restriction fragment length polymorphism (RFLP) of the Omp2b *locus* (79).

### Whole-genome sequencing and bioinformatics

Bacterial DNA from milk (Tt1) and kidney (Tt2) isolates was extracted and purified using the DNeasy Blood & Tissue Kit, according to manufacturer’s instructions (protocol “pretreatment for Gram-positive bacteria”) (Qiagen, Hilden, Germany) for WGS. Nextera XT DNA Library Preparation Kit was used according to the manufacturer’s instructions, and DNA was sequenced in the MiSeq Illumina platform.

Raw reads obtained were filtered out with Trimmomatic (80) for the removal of adaptors and low-quality raw reads. Genomes were assembled with SPAdes (81) using the reads that passed the quality control by FastQC. The quality evaluation of assemblies was performed using QUAST (82). MLST was performed to assign MLST profiles to assemblies by MLST software (T. Seemann, https://github.com/tseemann/mlst) and the public PubMLST repository (https://pubmlst.org/brucella/). The raw reads generated in this study were deposited in the European Nucleotide Archive under project PRJEB60581 (ERR11269022 and ERR11269021).

### Phylogenetic analysis

Sequence data from 14 *B. pinnipedialis* and *B. ceti* analyzed in other studies (14, 83) were included in the phylogenetic analysis performed in order to assess the genetic relatedness of the isolates described here relative to strains originating from other regions (Supplementary Data Set S1). *B. abortus* (GenBank Accession Numbers NC_006932.1 and NC_006933.1) and *B. melitensis* (GenBank Accession Numbers NC_003317.1 and NC_003318.1) were included in the analysis as outgroups. All sequences were mapped against the reference genome of *B. pinnipedialis* (GenBank Accession Numbers CP007743.1 and CP007742.1) using BWA (84) with default parameters. SAMtools (85) was used for sorting and compression of the obtained SAM files into BAM files. The variant calling was performed applying “mpileup” and “call” options with BCFtools (86). The resulting SNPs were filtered by removing those with a base quality <30 and a mapping quality <30. Consensus sequences were then created from the corresponding VCF (variant call format) file using BCFtools for each strain. Concatenated consensus sequences were used to generate a maximum likelihood phylogenetic tree using RAxML (87). The tree was constructed using the general time-reversible substitution evolutionary model with gamma correction and 1,000 bootstrap replicates. The tree was rooted using the sequence from *B. melitensis* and visualized using iTOL editor (88).

The number of SNPs between Tt1 and Tt2 strains was obtained from the VCF files to assess the differences between both strains.

### Serology

For the detection of antibodies against *Brucella* spp., an RBT was performed on serum samples from both animals using a *Brucella abortus* S99 suspension buffered to pH 3.6 (Spinreact, Girona, Spain) and a cell concentration of 3%.

In addition, both serum samples were analyzed using the INgezim Brucella Compac blocking ELISA (Ingenasa, Madrid, Spain), with *B. abortus* lipopolysaccharide (LPS) as the antigen. This type of ELISA has previously been used for the serological study of *Brucella* spp. in odontocetes (25, 30 – 33), and a 1/10 dilution has been recommended for the use of this kit in cetacean samples (31). Accordingly, each serum sample was diluted 1/10, and subsequently, the manufacturer’s instructions were followed. The seropositivity threshold was ≥40%, calculated according to the optical density (OD) using the following formula:


Percent inhibition(PI)=100×[1−(OD sample/OD negative control)]

## Data Availability

The raw reads generated in this study were deposited in the European Nucleotide Archive under project PRJEB60581 (ERR11269022 and ERR11269021).

## SUPPLEMENTAL MATERIAL

Supplementary Material S2 spectrum.01997-23-s0001.docx Brucella spp.-positive tissues in the molecular diagnosis and their relation with Brucella isolation and histological lesions observed.

Supplementary data set S1 spectrum.01997-23-s0002.xlsx Supplementary data set S1. Data of the sequences included in the phylogenetic study.

ASM does not own the copyrights to Supplemental Material that may be linked to, or accessed through, an article. The authors have granted ASM a non-exclusive, world-wide license to publish the Supplemental Material files. Please contact the corresponding author directly for reuse.
