# Supplementary material for: New insights into the pathogenesis and transmission of Brucella pinnipedialis: systemic infection in two bottlenose dolphins (Tursiops truncatus)
Source: Microbiol Spectr. 2023 Oct 6;11(6):e01997-23. doi: 10.1128/spectrum.01997-23 (PMC10848334; doi:10.1128/spectrum.01997-23)
Supplement: Supplementary Material S2 — Brucella spp.-positive tissues in the molecular diagnosis and their relation with Brucella isolation and histological lesions observed. [file spectrum.01997-23-s0001.docx]

**Supplementary Material S2. *Brucella* spp.-positive tissues in the molecular diagnosis and their relation with *Brucella* isolation and histological lesions observed.** NA: Not applicable; *: positive culture. Cycle threshold values of positive samples are indicated in brackets. The analysis were performed acording to a previously described real-time PCR targeting the insertion sequence IS*711* (73).

| **ID** | **PCR-Positive tissues** | **Histological lesions** |
| --- | --- | --- |
| Tt1 | Spleen (36.49) | NA |
|  | Urinary bladder (37.78) | In the connective tissue between muscle layers, some vessels were surrounded by small infiltrates of lymphocytes, histiocytes, and some neutrophils. The submucosa was lightly dotted with few lymphocytes. |
|  | Milk* (26.60) | NA |
| Tt2 | Cerebrum* (30.05) | Multifocally the meninges were markedly expanded by extensive infiltrates of macrophages, lymphocytes, fewer neutrophils, and plasma cells. Infiltrates predominated around the meningeal vessels. Multifocally and randomly in the neuropil, many vessels were surrounded by infiltrates of similar inflammatory cells, and astrocytes are numerous and hypertrophied. |
|  | Cerebellum (35.50) | NA |
|  | Spinal cord (30.16) | NA |
|  | CSF (27.96) | NA |
|  | Mesenteric lymph node (25.29) | NA |
|  | Pre-escapular lymph node (19.71) | The follicles were reactive and the sinuses filled with histiocytes and lymphocytes. Some follicles contained clusters of central hyaline material. |
|  | Pulmonary lymph node* (21.98) | It was composed of numerous follicles that contained large germinal centers, and the sinuses were dilated and full of lymphocytes, fewer histiocytes and some neutrophils. |
|  | Lung* (28.87) | In a multifocal and random manner, the parenchyma contained small to moderate-sized, perivascular, nodular aggregates of lymphocytes, macrophages, fewer neutrophils, and plasma cells. The alveolar interstitium was multifocally dotted with similar inflammatory cells. Many alveolar spaces contained a small amount of homogeneous, pale eosinophilic material with some intermixed foamy macrophages. Diffusely, the alveolar capillaries are markedly tortuous and filled with erythrocytes (congestion). |
|  | Liver (26.97) | In a multifocal manner, in the portal spaces and surrounding the centrilobular veins, there were nodular aggregates of lymphocytes, macrophages, fewer neutrophils, and plasma cells. The sinusoids were diffusely dotted with the same cells and moderately congestive. The Kupffer cells were hypertrophied. Many bile canaliculi were dilated and filled with bile (stasis), and the hepatocytes contained small, colorless, eosinophilic, ill-defined vacuoles. |
|  | Spleen (22.63) | Diffusely, the follicles were markedly enlarged and contained large germinal (reactive) centers. In the red pulp there was abundant extramedullary hematopoiesis, neutrophils and some macrophages loaded with hemosiderin. |
|  | Kidney* (28.51) | In the renal pelvis and rarely in the cortical interstitium, there were small foci of lymphocytes, macrophages, and neutrophils. |
|  | Urinary bladder* (26.33) | The submucosa contained small aggregates of mixed inflammatory cells that spread slightly between the epithelial cells. |
|  | Testicle (27.92) | Lacked observable histological alterations. |
|  | Epididymis (25.85) | Lacked observable histological alterations. |
